# Supplementary material for: Application Potential of Trichoderma in the Degradation of Phenolic Acid-Modified Chitosan
Source: Foods. 2023 Oct 5;12(19):3669. doi: 10.3390/foods12193669 (PMC10572696; doi:10.3390/foods12193669)
Supplement: Supplementary file 1 [file foods-12-03669-s001.zip › foods-2621886-supplementary.pdf]

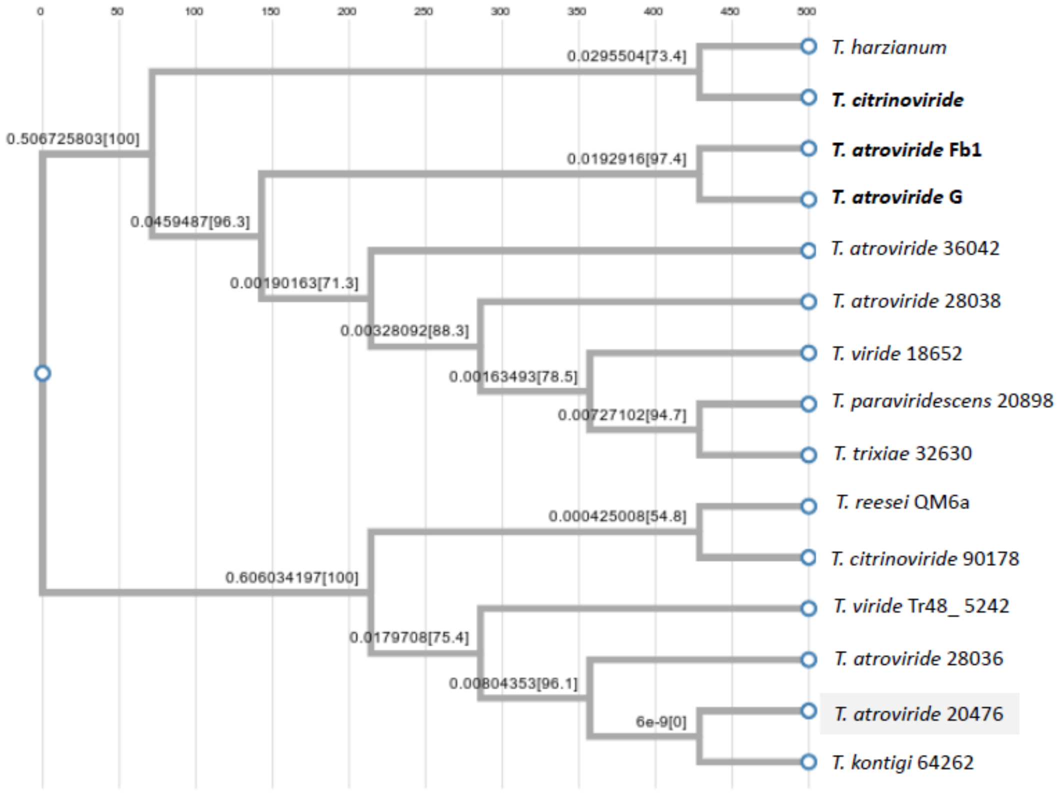

Figure S1. Dendrogram prepared using nucleotide sequences of the ITS region of the 18S rRNA gene and using Clustal W (https://www.genome.jp/tools-bin/clustalw). Sequences from NCBI's GeneBank were used to analysis: (Acc. no. OP586652.1) *T. citrinoviride*, (OP586653.1) *T. atroviride* G, (OP586643.1), *T. atroviride* isolate Fb1, (AY380909.1) T. viride strain ATCC 28038, (X93978.1) *T. viride* strain ATCC 18652, (AF279395.1) *T. citrinoviride* strain ATCC 90178, (JQ745264.1) *T. atroviride* strain ATCC 20476, (AF278796.1) *T. atroviride* strain ATCC 36042, (AF127152.1) *T. atroviride* ATCC 28036, (DQ315445.1) *Trichoderma trixiae* strain ATCC 32630, (NG_059411.1) *Trichoderma koningii* ATCC 64262, (DQ315434.1) *Trichoderma paraviridescens* strain ATCC 20898, (AF127154.1) *Trichoderma reesei* QM6a ATCC 13631, (AF127145.1) *T. viride* strain Tr48 ATCC 5242, (AY625068.1) *Trichoderma harzianum* strain NRRL 13019.
